# Effects of Unstable Conditions on Kinematics and Performance Variables in Young Handball Players

**DOI:** 10.1515/hukin-2015-0032

**Published:** 2015-07-10

**Authors:** Tomás Urbán, Óscar Gutiérrez, Francisco J. Moreno

**Affiliations:** 1 Sport Research Center. Miguel Hernández University of Elche, Elche-Alicante (Spain).

**Keywords:** variability, kinematic, adaptation, handball

## Abstract

The execution variability and outcomes found in throwing actions have received special attention in numerous studies in recent years. The aim of this study was to analyze the effect of an unstable condition on velocity, accuracy and kinematics of movement in the seven metres throw in handball. Twenty-five young handball players took part in an experiment, throwing towards a target on a stable and an unstable surface. Each participant performed 32 throws, 16 for each situation. Linear variability of the dominant hand was assessed by 3D Motion Tracking. A radar sports gun was used to record the velocity of the ball and the throws were video recorded to establish their accuracy. Results showed significant decreases in throwing velocity in unstable conditions, but these did not significantly affect the accuracy achieved in performance. Differences were also found in movement kinematics between the two throwing conditions and relationships were found between kinematics, velocity and accuracy.

## Introduction

Handball is a very strenuous body-contact team sport placing heavy emphasis on running, jumping, and throwing (Toyoshima et al., 1976). In addition, playing conditions constantly change during the development of the game. Usually, an adversary is located between the thrower and the goal, causing the throw to be performed in varied conditions, forcing the thrower to adapt to situations of instability that result in great variability both in the movement’s execution and in the outcome of the throw. Some previous studies have shown the influence of instability caused by the opposition in throwing kinematics (Gutierrez-Davila et al., 2006) or in outcome variables such as throwing velocity (Rivilla et al., 2010), what has also been studied in overhand actions in other sports (Vila et al., 2009).

Therefore, the maintenance of stability is a fundamental motor act in handball, as well as in many other motor actions in the upright standing posture. Stability provides the basis for locomotion and most other movement tasks (Vaillancourt and Newell, 2002). The system regulates the body’s postural sway through the complex interaction of somatosensory, visual, and vestibular sensory feedback networks, numerous brain regions, and the musculoskeletal system (Winter et al., 1990; Palmieri et al., 2003; Manor et al., 2010). In this sense, as we have noted, the opposition of an adversary causes continuous unstable conditions, forcing the player to adjust the movement during the execution of the throw. Highly skilled athletes have shown higher capacity to adapt to changing environmental conditions with the aim of improving performance (Scholz et al., 2000; Wilson et al., 2008). During their careers, skilled athletes have experienced continuous variable situations of instability during training and competition. Practicing in increased variability situations during the performance of a specific task has been proposed as an adequate way to enhance performance and the ability to adapt (Davids et al., 2004). In recent years, several studies have outlined the importance of variability in motor control and learning (Barttlet et al., 2007; Button et al., 2003; Miller, 2002; Robins et al., 2006). From this perspective, movement variability players a functional role, providing the nervous system with a more effective environment exploration and, therefore, with an adequate adaptation to different changing environmental conditions (Schöllhorn et al., 2009; Phillips et al., 2010). In line with this, several studies have argued that functional variability would allow the athlete to cope with perturbations due to environmental or task constraints (Schorer et al., 2007; Wilson et al., 2008).

The adaptation process to unstable conditions in handball throwing influences the kinematics of movement and, therefore, accuracy and ball velocity. The relation between both variables (accuracy and velocity) has been analyzed frequently in team-handball because, as in many rapid-aimed movements, the efficacy criterion requires balance between speed and accuracy of movements (Bayios et al., 2001; Fradet et al., 2004; Gorostiaga et al., 2005; van den Tillaar and Ettema, 2003, 2006; Wagner et al., 2011). It has been traditionally argued that an increase in velocity causes a decrease in accuracy, but Plamondon and Alimi (1997) presented a critical review literature dealing with speed/accuracy trade-offs in rapid-aimed movements. Recent results have shown that increases in throwing velocity do not necessarily lead to a loss of accuracy (García et al., 2013). Van den Tillaar and Ettema (2003, 2006) noted that a decrease in throwing velocity in handball players does not noticeably improve accuracy.

In this study, we analyzed the characteristics of team handball throwing and the effects of unstable conditions on movement kinematics and performance variables (speed and accuracy). We asked handball players to carry out throws towards the goal on an unstable surface with the aim of inducing the player to modify throwing kinematics by adapting to changing conditions. The aim of this study was to analyze the effects of unstable conditions on handball throw kinematics, throwing performance (velocity and accuracy), and its relation to movement variability.

## Material and Methods

### Participants

Twenty-five right-handed handball players, all of them males with an average age of 17.9 ± 3.7 years, took part in this study. These players had at least six years handball practice and a training frequency of five sessions per week plus a competition day (a match day). All participants took part in different national competitions in Spain. In accordance with the ethical guidelines of the Miguel Hernandez University, all participants signed informed consent forms before the research began.

### Procedures

**Figure 1:**
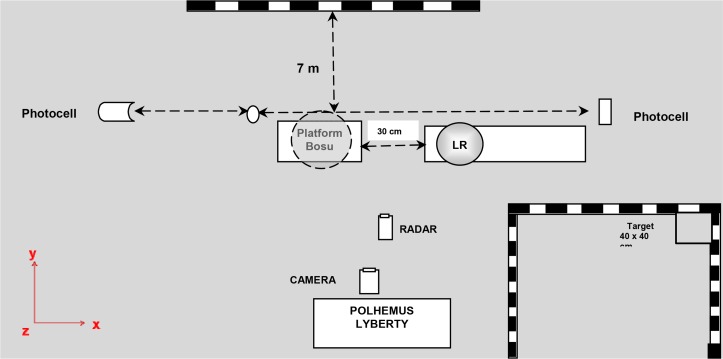
Experimental set.

Participants performed a specific warm up before the test. Afterwards, they performed four series of eight throws at seven metres distance from the goal. The throws were performed in a standing position with feet placed at shoulder width (30 cm between heels), and foot orientation was such that the vector formed by the heels was in a parallel position to the medial-lateral axis of both platforms (Caballero et al., 2013). Two of the four series were performed on a stable surface (floor) and the other two series were performed on an unstable surface (standard BOSU® balance trainer). The feet position during throwing was unchanged in both situations. To avoid progressive error, the series were counterbalanced and randomly assigned. The participants were asked to throw a standard handball (IHF size 3) at a target (40 cm × 40 cm square) located in the top right hand corner of the goal (Figure 1). To avoid fatigue effects on velocity and accuracy, the participants rested for five seconds between each throw and rested for five minutes between series. The number of throws performed should allow the assessment of velocity and accuracy characteristics due to the stability of the throw pattern in the specialization of the players (van den Tillaar and Ettema, 2003b). The instruction given to the participants was to “throw at the highest velocity and with the highest accuracy possible towards the target”.

In every throw, the ball was previously located on a square platform 1.40 m on the left side of the participant. Players were asked to stand comfortably with both feet pointing towards the goal, to grasp the ball with their right hand, with no help from their left hand when throwing towards the target.

Kinematic variables of the dominant hand, arm and hip movements were registered by a three-dimensional electromagnetic position tracking system (Polhemus^TM^ Liberty), with accuracy of 0.076 cm for position (anterior-posterior, medial-lateral and vertical axis) and 0.15° for angular orientation (Azimuth, Elevation and Roll) with 240 Hz sampling frequency. The sensors were located on the back of the dominant hand (metacarpus medial part), arm (impression or deltoid tuberosity and iliac crest).

An infrared light photocell allowed us to detect when the player initiated the start of the throw. To measure the throwing speed, a radar gun was used from Sports Radar Ltd (model SR 3600) with ± 0.44 m/s sensitivity. A digital camera, SONY DCR-HC18E (50 Hz sampling frequency), was placed opposite the goal at 9 m from the goal line at a height of 2.5 m. The centre of the ball when the ball entered the goal was digitalized with Visual Basic 5.0 application software developed in the laboratory, which identified the deviations of throws with respect to the centre of the target. The Matlab 7.11 routine was used for the calculation of real-space Cartesian coordinates.

### Measures

To estimate the effect of unstable conditions on throwing, velocity and accuracy of the ball as well as the kinematics of the dominant hand were measured.

Mean radial error (MRE), and absolute error in the vertical (y) and lateral (x) axis were used to measure accuracy. Mean Radial Error was measured as the average of absolute distance to the centre of the target. Thereby, we considered the centre of the square as the target point.

Kinematic variables of the dominant hand were analyzed from the moment that the throwing motion started until the end of the throw. Time zero was taken when the participant picked up the ball located on a square platform (marked by photocell). Considering that the kinematic parameters of the hand position were affected by the use of unstable surfaces of greater height (30 cm) and the anthropometric characteristics of each participant, the spatial data of the dominant hand were taken into account relatively to the range of motion. To perform the analysis between situations, the values of velocity were used. The overall speed of the executing hand and the instantaneous velocity of the hand at the time when the ball was finally released from the hand were analyzed in the anterior-posterior, media-lateral and vertical axis.

Variability in accuracy was measured as the standard deviation of MRE (variable error). To capture relevant information about variability of throwing over time, we measured hand movement variability through a continuous method (James, 2004; Hamill et al., 2000). Data from hand velocity were normalized to 1000 data points and aligned at the beginning of the movement. After normalization, we calculated the mean and standard deviation point by point of the test series for each participant (equations 1a and 1b). From these data the average of the typical deviation of all the data series (equation 1c) (Winter, 1984) was calculated, obtaining an estimate of the variability of the trajectory of the hand.

### Statistical Analysis

Out of the 25 participants who took part in this study, six participants were excluded from the kinematic analysis due to problems with regard to collecting data with the instrumentation used. Therefore, analyses were performed for the accuracy and velocity of the 25 participants and for the kinematics of 19 participants. The reliability analysis between measures showed ICC scores larger than 0.8 for all variables (Atkinson and Nevill, 1998; Schabort et al., 1998; Vincent, 1994). Effect size (ES) was calculated represented by d and interpreted for a recreationally trained sample according to Rhea (2004) as d<0.35 (Trivial), 0.35<d<0.80 (Small), 0.80<d<1.50 (Moderate), and d>1.50 (Large). A Kolmogorov-Smirnov test was conducted to test the data’s deviation from the normal distribution. Repeated measures ANOVA were carried out to compare the effects of the stability conditions. Pearson correlations were performed to analyze the relationship between kinematic variables, movement variability and performance in the seven metres handball throw.

## Results

**Figure 3:**
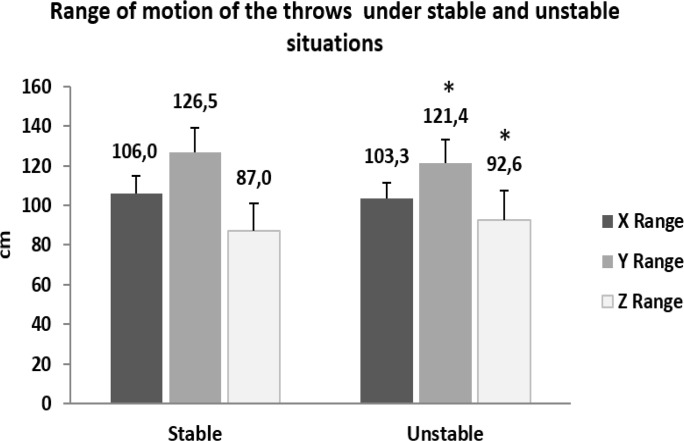
Comparison of the range of movement in all three axes in both throwing conditions. * Significant difference between conditions (p <0.01).

The results obtained by the effect of the unstable condition on the kinematic variables of the throwing movement showed a significant decrease in the range of movement in the anterior-posterior axis (t 18.1 = 4.020; p<0.01; d −.41) and a significant increase in the range of movement in the vertical axis (t 18.1 = −4.723; p<0.01; d .41) in the unstable condition. Also, significant and longer movement durations were found in the unstable condition (t 18.1 = −2.361; p<0.05; d .42) (Figure 3).

The analysis of kinematic variables in the moment of the ball release showed significant differences in the anterior-posterior position of the arm (t 18.1 = 2.802; p<0.05; d −.49) and the hip (t 18.1 = 2.778; p<0.05; d −.35), showing lower values in the unstable condition. Additionally, a reduction of the peak velocity of the hand (t18, 1 = 2.884, p=0.01; d −.45) and the hip (t 18, 1 = 3.194, p=0.01; d −.46) was found in the unstable condition. The decrease in hand peak velocity was significant in the anterior-posterior (t18, 1=2.870; p=0.01; d −.38) and vertical axis (t 18, 1=2.493; p<0.05; d −.25). The time to reach the maximum velocity also increased, showing longer time to peak velocity in the hand (t 18, 1 = −2.450, p<0.05; d .43) and the hip (t 18, 1 = −2.493, p=0.01; d .45) in the unstable condition.

The variability of movement during throwing in the unstable condition was increased only in the position of the hip, with significant increases in the medial-lateral (t 18, 1 = −2.592, p<0.05; d .84) and anterior-posterior axis (t 18, 1 = −2.699, p<0.05; d .57). However, no differences were found in variability between conditions in the arm or hand position, nor in velocity variables.

**Figure 4:**
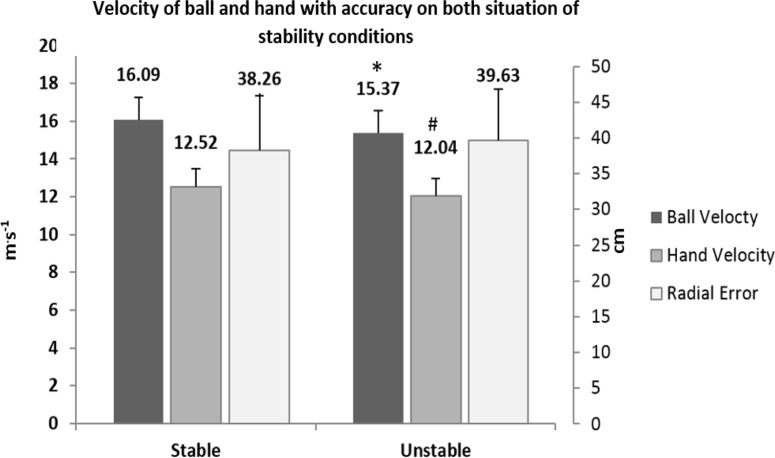
Comparison of ball velocity, hand velocity and radial error on both stable and unstable conditions. * Significant difference between conditions (p <0.01). # Significant difference between conditions (p<0.05).

The velocity of throwing was affected by the unstable condition, significantly reducing the ball velocity of the throws when these were performed in the unstable condition (t 24, 1 = 5.738, p<0.01; d −.47) (Figure 4), but no significant differences in accuracy were found.

There were differences regarding the relationship between velocity and accuracy depending on the experimental condition. In the stable condition, the correlation analysis performed for each participant did not show a clear relationship between velocity and accuracy. Only two of the 25 participants showed an inverse relationship between velocity and accuracy. The rest showed no evidence that throwing at higher velocity induced worse results on accuracy. When the participants threw on the unstable surface, a positive correlation between the duration of the movement and the radial error (r=.643; p=0.003) was found. In the same way, a positive correlation was found between the range of movement in the vertical axis and radial error (r=.444; p=0.028). Thus, players who performed a temporally briefer and spatially shorter movement obtained a smaller error. The duration of the movement in the stable condition showed a significant correlation with the radial error in the unstable condition (r=.456; p=0.05), suggesting that players with shorter movements in the stable condition showed higher accuracy in the unstable condition.

Averaging the values of throwing velocity and accuracy from all the participants, no clear inverse relationship was found between these variables. In the stable condition, we only found a positive correlation between the velocity of the hand in the medial-lateral axis and absolute error in the lateral axis (r=.713, p=0.001). This correlation was also observed with constant error in the lateral axis (r=−.549; p=0.015), indicating a lateral deviation to the left of the target in higher velocity throws. In the unstable condition, it was found that a higher velocity of movement was related to higher accuracy in throwing, with a negative correlation between velocity of the dominant hand and the radial error (r=−.474, p=0.040). Decomposing velocity values by axis, the relationship appeared mainly in the anterior-posterior (r=−.550, p=0.015) and vertical (r=−.654, p=0.002) components of velocity. Therefore, participants with higher movement velocity in the anterior-posterior and vertical axis showed better accuracy values.

## Discussion

This work was conducted to study the effect of an unstable surface on throwing kinematics and performance variables (velocity and accuracy) and how this is related to the variability of performance.

The results showed a modified pattern of throwing when players threw in unstable conditions. In sport situations, instability is normally caused by the effect of the opposition. During play, the opposition of an adversary continually forces a player to adjust their movement during the execution of the throw (Rivilla et al., 2010; Gutierrez-Davila et al., 2006). In this sense, throughout their careers, players are constantly faced with situations of instability during training and competition. The results seem to suggest that players use different strategies in the throws during unstable conditions, showing that shorter movements in throwing would be more effective to adapt to unstable conditions. The unstable condition modified the throwing pattern, reducing the velocity of the hand and the hip, while increasing arm velocity, negatively affecting the proximal-to-distal sequence (van den Tillaar and Etemma, 2009; Wagner et al., 2010b). The lower velocity of throwing in the unstable condition can be related to an altered progression of limb segmental motion from proximal to distal, based on the principle of optimal profit from the reactive forces between segments when producing maximal speed of the distal segment (Herring and Chapman, 1992). In this way, timing to reach the maximal linear velocity in limb segmental motion was affected by the unstable condition, showing an increase of this variable in the hand and the hip. These results agree with those obtained in the velocity of body segments implicated in throwing, where the velocity of the segments was reduced (Wagner et al., 2011).

The overall variability of movement and particularly the variability of the arm and the hand during throwing were not affected by the unstable surface. Nevertheless, increased variability in the anterior-posterior and medial-lateral axis of the hip was found. This proximal variability could help to reduce distal variability, and would be related to an adaptation strategy. In a deeper analysis of these results, we noted that this difference was only significant in the best participants in the sample (those with lower error on the stable surface). This can suggest an enhanced variability strategy to maintain performance. Handball players would offset the increased variability in the lower body, releasing degrees of freedom that would allow them to maintain consistency in the execution of the upper body (Scholz et al., 2000; Wilson et al., 2008). In view of the results, the participants would explore and search coordination structures, freezing and releasing degrees of freedom to perform the throws effectively under particular task conditions (instability of the surface or support) that allow an optimal performance (Newell, 1985). Thus, skilled players have shown higher capacity to adapt to changing environmental conditions to improve performance (Scholz et al., 2000; Wilson et al., 2008). Thereby, movement variability plays a functional role, allowing a more effective environment exploration and, therefore, with an adequate adaptation to different changing environmental conditions (Schöllhorn et al., 2009; Phillips et al., 2010), allowing the players to compensate for the increment of movement variability during throwing (Wagner et al., 2011).

The velocity of throwing was significantly reduced when the throws were performed in the unstable condition. Regarding the velocity of the ball, the results showed that despite the reduction in velocity caused by throwing in an unstable condition, the players that threw faster in stable conditions also threw faster in unstable conditions. These results are in line with different studies in which more experienced players threw at higher velocity than the less experienced players, regardless of the demands of the task (Bayios et al., 2001; Gorostiaga et al., 2005; Wagner et al., 2012). However, accuracy results, depending on whether the throws were performed in unstable or stable conditions, were similar for both situations, indicating that both the change in the motor control strategy and the reduction of velocity allowed participants to maintain the accuracy unaffected by throwing conditions.

The results of the relationship between velocity and accuracy agree with findings of previous works comparing the effects of instruction on trade-off velocity-accuracy in different levels of performance (novices versus experts) (Van den Tillaar and Ettema, 2003, 2006; García et al., 2013). Previous results found that throwing velocity did not decisively affect the accuracy, arguing that expert players could maintain high levels of accuracy regardless of the velocity of throwing.

The positive correlation found between radial error with both duration of throwing and the range of movement on the vertical axis, seems to indicate that players with shorter and briefer movements show a lower affectation of the instability effects on accuracy. This could be due to the high levels of specialization of players caused by the larger amount of practice, having adapted to specific situations of instability during throwing (García et al., 2011) and showing a strategy based on the reduction of both amplitude and movement time to reach the target.

On the other hand, the results on instability conditions showed a positive relationship between hand velocity in the medial-lateral axis and absolute error in the lateral axis. This would indicate that players with higher hand velocity in the medial-lateral axis are less accurate. However, the inverse relationship between medial-lateral velocity and constant error notes an accuracy bias to the left of the target. The greater the medial-lateral velocity of the hand, the more left error bias. This could be due to the fact that the ball is released during an internal rotation of the shoulder, forearm pronation and wrist flexion in a proximal-distal performance sequence (participants were all right-handed) (Wagner et al., 2012). In the unstable condition, the compensation of internal rotation is hindered, increasing the amount of error in the lateral axis.

In the unstable condition, the results of correlation values showed an inverse relationship between the velocity of movement and radial error, suggesting that the participants increased the velocity and reduced the movement time in order to be more accurate. This is in line with the results obtained in previous studies where skilled players were able to maintain velocities of throwing approaching their maximum and, in turn, to optimize accuracy (van den Tillaar and Ettema, 2004).

The kinematic analysis showed that throwing in unstable conditions substantially modified the technical gesture in order to adapt to the new condition without significantly affecting accuracy. Nevertheless, the opposite occurs regarding the velocity of the throw, decreasing significantly when throws are performed in unstable conditions.

The players showed an ability to adapt to the increased variability caused by the instability of unstable conditions, performing the throws with the same effectiveness as in stable conditions. In order to maintain effectiveness, the players increased the velocity of the movement and reduced their movement time. The presence of unstable situations in handball throwing task during training sessions is recommended to facilitate faster and shorter movements and to improve the efficiency of the throws. The relationships found in this study may contribute to a better understanding of how the unstable conditions that occur during a game can affect throwing kinematics and performance variables such as velocity and accuracy.

## Figures and Tables

**Figure 2:**
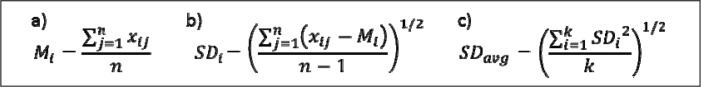
Equation 1
